# Importance-Performance Matrix Analysis (IPMA) to Evaluate Servicescape Fitness Consumer by Gender and Age

**DOI:** 10.3390/ijerph17186562

**Published:** 2020-09-09

**Authors:** Jerónimo García-Fernández, Jesús Fernández-Gavira, Antonio Jesús Sánchez-Oliver, Pablo Gálvez-Ruíz, Moisés Grimaldi-Puyana, Gabriel Cepeda-Carrión

**Affiliations:** 1 Department of Physical Education and Sport, Universidad de Sevilla, 41013 Sevilla, Spain; jeronimo@us.es (J.G.-F.); mgrimaldi@us.es (M.G.-P.); 2 Departamento de Motricidad Humana y Rendimiento Deportivo, Universidad de Sevilla, 41013 Sevilla, Spain; sanchezoliver@us.es; 3 Máster Universitario en Gestión Deportiva, Universidad Internacional de Valencia, 46002 Valencia, Spain; pablo.galvez@campusviu.es; 4 Management and Marketing Department, Universidad de Sevilla, 41018 Sevilla, Spain; gabi@us.es

**Keywords:** importance-performance matrix analysis (IPMA), fitness industry, sport consumer, servicescape, gender, age

## Abstract

The fitness sector has always been linked to the analysis of the loyalty of its consumers. Different studies have shown the importance of sports service and human resources for greater customer loyalty. However, few works have studied how the physical environment or servicescape influences the behavior of consumers in fitness centers based on gender and age. Therefore, the objective of the study was to analyze the relationship between servicescape and the loyalty of fitness center consumers, analyzing through the Importance-Performance Matrix Analysis (IPMA) what the aspects to improve according to gender and age are. The sample was 10,368 fitness center customers (5864 women and 4504 men). After the IPMA, it was concluded that the main improvement margins in general in fitness centers were the equipment and the facility condition, and the facility layout. In turn, in relation to gender and age, the aspects with room for improvement were to a greater extent for equipment and facility condition in women over 21 years of age, and in facility layout for women between 21 and 40 years old and 51–60 years old. Regarding men, the aspects with the highest performance margins were the equipment and facility condition in all the age groups, the facility layout in men up to 50 years old, and the signage in men up to 40 years old and from 51 to 60 years old.

## 1. Introduction

The fitness sector is a rapidly growing industry [1]. According to the International Health, Racquet, and Sportsclub Association [2], there has been a significant growth in the last decade in terms of the number of gyms, fitness clubs, and other fitness centers, as well as the amount of people who join them. Although at a slower rate than in previous years, the fitness market continues to grow and be consolidated in Europe and Spain [3]. Precisely, the Spanish fitness sector in 2019 increased its membership by 3.3%, with a penetration of this activity of 11.7%. This has meant 2.352 million euros in terms of income, meaning the fifth position in the ranking of European countries in this regard and continuing its upward trend in the fitness sector. Observing the evolution of recent years, it can be said that the Spanish fitness sector is in a state of maturity, but still with room for growth [3].

Despite the growth of the fitness industry, the current situation in the sector makes stakeholders increasingly concerned about the good performance of their organizations in order to determine priorities and/or needs, and to formulate better strategies [4,5]. In this way, the fitness industry immersed in the service sector, has been focused on the value proposition of its offer, which is ultimately intangible. Yet, tangible aspects such as the space where the sports service is produced cannot be hidden and could have a strong effect on the perceptions of consumers themselves [6]. From this perspective, the servicescape concept emerges, which is understood as “the physical environment that impacts the behavior of customers and employees in service organizations” [7]. In fact, it is understood that both customers and employees perceive the physical environment through a variety of objective environmental factors and that both groups can respond cognitively, emotionally, and physiologically to this tangible environment [6]. Precisely, the fitness sector has realized the importance of the physical environment to improve the quality of its service, having hardly analyzed the servicescape in fitness centers [8,9].

Studying this is essential in fitness centers, since their knowledge and their proposals for improvement can have an impact on the increase in the number of users and, above all, on customer loyalty [8]. Therefore, if the consumer perceives the aspects of servicescape as positive, they will have a greater chance of being a loyal customer [10]. In turn, gender or age could be presented as determining variables in the perception of the different aspects that make up the servicescape, due to their differences in the perception of sports services [11,12].

In light of these considerations, one of the most useful tools for directors and managers is the importance-performance analysis, which starts from the modeling of partial least squares structural equations (PLS-SEM). This analysis, called importance-performance matrix analysis (IPMA) [13], is a useful technique to identify the areas of improvement that management activities must address [14], and which has hardly been used in studies of the fitness sector. Therefore, this analysis would help to understand the influence of servicescape on consumer loyalty and would identify those aspects with room for improvement by gender and age. The findings would help managers to propose improvement actions segmented by gender and age to achieve more loyal consumers.

Based on the above and despite the high customer turnover in the fitness industry and its growing global importance [4], there is a research gap regarding the relationship between servicescape and consumer loyalty in fitness centers. Similarly, although there are studies in the fitness sector that have analyzed the importance and performance of fitness consumer [12], there is also a breach in the sports management literature analyzing through IPMA the variables servicescape and loyalty according to gender and age. Based on these limitations in the academic literature, the research questions are: Is there a relationship between servicescape and consumer loyalty in fitness centers? Moreover, what are the aspects of servicescape that can be improved in fitness centers from the point of view of the consumers’ gender and age? Based on the comments and answering to the research questions, this study aims to analyze the relationship between servicescape and the consumers’ loyalty of fitness center, analyzing through the IPMA what the aspects to improve according to gender and age are.

## 2. Theoretical Foundation

### 2.1. Servicescape and Its Relationship with Consumer Loyalty in Fitness Centers

Servicescape is considered the design of service environments made up of environmental dimensions such as space and its function, signs, symbols, instruments, and the people who interact in the service delivery environment. In this sense, the concept understands space as holistic, understood as a whole [15]. That is, “everything that is physically present around the consumer during the service encounter transaction” [16]. In fact, servicescape is a vital concept to know and intervene in customer behavior. Hence, it is considered a facilitator that adds or hinders the behavior of both employees and customers when carrying out their activities in the service sector [17,18]. For this reason, service providers must offer environments that enhance consumer experiences and facilitate operational ease and promote overall business efficiency [19].

The study of the servicescape has been carried out in different contexts, such as banks [20], healthcare settings [17,21]; game rooms [22,23], travel [24] and sports environment [25,26]. Although there are several articles focused on the sports context, few have focused on examining the servicescape in the environment of fitness centers [8,9]. Thus, since fitness centers are highly dependent on their physical environment, it is essential to understand the impact of the physical environment on users and their behavior, obtaining a better understanding of environmental concepts and what factors could influence consumers’ perceptions.

If these perceptions had a positive influence on customer behavior, consumer loyalty would be increased. This loyalty is desired by fitness centers which, due to continuous competition, has led them to find ways of differentiating themselves to retain existing members and attract new ones [1]. A solution could be linked to servicescape. In fact, Chang (2016) examined a service firm and identified a positive relationship between the servicescape and customers’ behavioral intentions [27]. Others, such as Madzharov, Block and Morrin (2015), also examined how ambient scents affect consumers’ perceptions and feelings and, subsequently, their purchase intention [28]. Recently, the study of Tran, Dang and Tournois (2020) has revealed the positive relationship between servicescape and consumer loyalty in coffee stores [29], as also indicated by the studies of Lee, Fu and Tsai (2019) and, Rai and Anirvinna (2019) [30,31]. Regarding studies that have analyzed both variables in the sports sector, Hightower, Brady, and Baker (2002) showed that there was a positive relationship in sporting event customers [32]. In particular in the fitness sector, only one study is found that has confirmed the positive relationship between servicescape and loyalty [8], although the study had the limitations of a small sample and a limited number of servicescape dimensions.

For all this, taking into account that loyalty is one of the main objectives of the fitness sector [33], different studies have analyzed what could be the variables that would influence customer loyalty in the fitness sector [34,35,36]. Nevertheless, although the literature has shown that there is a positive relationship between servicescape and consumer loyalty, there is a gap in the sports management literature that has examined the relationship between servicescape and loyalty.

### 2.2. Differences in the Perception of Sports Services by Gender and Age

Providing information to sports organizations about the relative proportion of users with a certain profile, what their sociodemographic characteristics are, as well as their perception and consumer loyalty, allows improving the design of the service offer and therefore improving consumer satisfaction [11].

Despite the existence of research in the field of fitness centers on consumer perception [37,38,39], most is focused on demonstrating the relationship between antecedent variables of fidelity, without having examined whether these relationships differ according to gender and age, even though they are extremely important variables [11].

Thus, although there are few contributions in this regard, some studies have shown that women have more positive perceptions and that they in turn have better fidelity indexes [40,41,42]. Notwithstanding, recent studies, such as that by León-Quismondo et al. (2020), have indicated that dissatisfaction was greater in women [12].

On the other hand, age is one of the sociodemographic variables that can most condition the behavior or perception of clients in fitness centers. The large number of sports services and types of clients has generated the need to carry out studies that help focus an offer according to the existing demand in the fitness centers [43,44]. Specifically, studies such as that of Yu et al. (2014) point out that age is directly related to the importance given to both the type of physical activity that is practiced in these centers and the aspects that are important for these people during their consumption [45]. For example, older people have a greater inclination for activities that provide them with healthy benefits with which to face their day-to-day life compared to younger people, whose centers of interest are more varied, challenging health with exercise that will allow them to achieve a more esthetic body [46,47]. For this reason, directing the marketing strategy toward one public or the other, or achieving loyalty to both, are very delicate and important decisions that managers must face in their professional performance. In fact, perhaps due to the orientation that each age group has to physical activity, it has been shown that older people give less importance to superfluous aspects of the facilities, compared to other age groups (young and young adults) who do [47]. Finally, almost all the population groups agree on the importance of having pleasant monitors and the accessibility of the center itself as fundamental factors in fidelity [47,48].

### 2.3. Important-Performance Matrix Analysis

Strategic management of service perceptions and the physical environment of sports spaces are very important for success in sports organizations [49]. For this reason, as has been shown, studies analyzing consumer perceptions in the fitness industry are increasingly more frequent [1,12].

One of the tools to assess consumer perceptions and position where managers should direct their efforts is the importance-valuation analysis (IPA), published by Martilla and James (1977) [14]. In fact, in the academic literature on sport management, there are some studies that have used it, providing what aspects must be improved for a better service [12,50,51]. The simplicity of its use and its applicability to explain customer satisfaction and suggest management strategies on how to prioritize resources makes this methodology very attractive for use in sports management [52]. Despite its simplicity and application, the IPA analysis offers a series of shortcomings that question its validity and usefulness due to the lack of standard criteria, along with conceptual and methodological issues [13,53].

For this reason, looking for an approach from a more global perspective, the importance-performance matrix analysis (IPMA) emerges. This analysis can allow directors and managers to improve their management strategies, since it points out the main factors that require an immediate response [54]. The IPMA allows for prioritizing constructs to improve a certain target construct, identifying the most important areas for specific actions. The findings provided by the IPMA are important in practical studies that identify the different impacts that certain dimensions of the construct have on the phenomena [13]. The decision to apply this technique is supported by its usefulness in other sectors (Table 1).

In addition, its application is based on three motivations: IPMA facilitates more rigorous management decision making (1), IPMA is a powerful tool that can help managers set better priorities and better allocate scarce resources (2), and IPMA has guidelines for performance evaluation that serve both a company and the people who invest in it (3) [65]. In other words, IPMA provides a better understanding of where management should focus its attention. However, although IPMA has been used in different sectors helping with the findings to better management [13], so far it has not been applied in the fitness sector.

**Figure 1:**
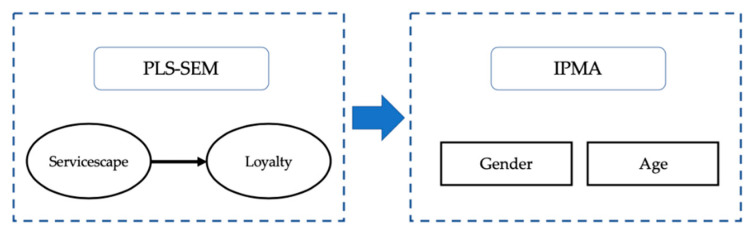
Theoretical framework of the study. IMPA: Importance-Performance Matrix Analysis; PLS-SEM: Partial Least Squares Structural Equations.

Based on the previous literature and responding to the objectives of the study, Figure 1 exemplifies the theoretical framework.

## 3. Materials and Methods

### 3.1. Participants

Participation was completely voluntary. We collected data for this study by using the convenience sampling method from November 2018 to March 2019. We distributed forms to 56 low-cost Spanish fitness centers. After describing the study’s purpose and significance, the people who agreed to take part completed the survey. The sample was 10,368 fitness center customers. 56.55% of the respondents (*n* = 5864) were women and 43.44% (*n* = 4504) were men. In terms of age, 3.74% (*n* = 388) were less than 20 years old, 26.82% (*n* = 2781) between 21 to 30 years old, 28.65% (*n* = 2970) were 31 to 40 years old, 26.52% (*n* = 2750) were 41 to 50 years old, 11.27% (*n* = 1168) were 51 to 60 years old, and 3.00% (*n* = 311) were aged over 60 years. In terms of period of time the respondents had been enrolled and had exercised at the center, 46.1% (*n* = 4789) of the participants had been enrolled for less than a year, 25.7% (*n* = 2668) had been enrolled for more than a year and less than two years, and 27.2% (*n* = 2911) had been enrolled for more than two years. Table 2 shows data from the sample regarding weekly frequency, previous fitness experience, and training prescription.

For this study it has not been experimented with humans or animals, nor has any vulnerable group been used in an inadequate way, all the data obtained was done through anonymous online surveys of 10,368 adults who were asked to fill out a questionnaire confidentially and to be honest with their answers, they were informed that the data obtained would be processed and published for the improvement of the fitness sector and that any personal information that they provided would be treated anonymously. To which they gave their express consent, without making it necessary for these studies to be evaluated by an external ethical committee for approval, since it has not affected in any way the ethical principles for research with humans contained in the Declaration of Helsinki.

### 3.2. Instruments

The instrument used to measure the variables of this study is a structured questionnaire. All the variables included in the study were measured with multi-item scales validated by other researchers, using a Likert scale ranging from 1 to 7. 21 items were used for servicescape were based on Kim, et al. (2016) [9]. The future intentions were measured using three items developed by Zeithaml et al. (1996) [66]. This measure has been used in other studies to measure customer loyalty in the fitness industry [35]. To adapt the study to the Spanish context, the scales of servicescape and future intentions went through a process of translation and synthesis to ensure that there were no errors of interpretation. To that end, the procedures were followed in accordance with Martín-Consuegra et al. (2015) [67]. Based on this, it is possible to compare and examine the equivalence and precision of the translated questionnaire.

### 3.3. Procedure

The chains of low-cost fitness centers with more than five sport facilities were contacted according to data from [68]. These fitness chains added a total of 213 low cost fitness centers, of which 56 low cost fitness centers decided to participate. The objectives of the study, the instrument, and the practical implications were explained to the managers of the fitness centers. Finally, five fitness center chains chose to participate, with a total of 56 fitness centers. Data collection took place after obtaining permission from the administration of each participating center. The participants gave their consent. Voluntary participation and the confidentiality of their responses was assured. Data collection was carried out through an online questionnaire using Google Forms over a period of five months. The participants took approximately 10 min to complete the questionnaire.

### 3.4. Data Analysis

All our measures were operationalized as composites [69,70]. Therefore, we decided in favor of using PLS-SEM as the best data analysis tool. All the composites were estimated in Mode A because the indicators that compound composites are correlated. Our study adopted both an exploratory and a predictive approach following Cepeda-Carrión et al. (2019) [71]. To assess models using IPMA with PLS-SEM an additional procedure implemented by SmartPLS (Version: 3.2.9; SmartPLS GmbH Company, Bönningstedt, Germany) has been established [72]. Additionally, we test our path model using the classical two step assessment proposed by Hair et al. (2014): (1) the assessment of the measurement model and (2) the assessment of the structural model [73]. In order to find out the significance of parameters, we used a bootstrap procedure [74]. Bootstrapping is a resampling procedure that is able to determine the significance of: path coefficients and the weights and loadings of the indicators for each composite (i.e., the latent variables).

To detect potential issues of common method bias (CMB) due to the instrument used, a full collinearity test based on variance inflation factors (VIFs) was carried out. According to Kock and Lynn (2012) [75] when a VIF achieves a value greater than 3.3, there would be an indication of pathological collinearity. This would warn if a model may be contaminated by CMB. The present model, with a maximum VIF of 1.02, may be considered free of CMB.

## 4. Results

According to the assessment of the measurement model, our outcomes show that the measurement model meets all the requirements suggested by Hair et al. (2014) [73]. First, the individual items are reliable because all the standardized correlation weights exceed 0.7 (Table 3), except one indicator. Yet, as this does not affect the rest of the indices of reliability and validity, we decided to keep it in our analysis. Second, because consistent measures for all the composites are greater than 0.8 (Table 4). These measures (composite reliability, Cronbach’s alpha and Dijkstra-Henseler´s rho) are suggested by Hair et al. (2019) [76] and Henseler et al. (2016) [77]. Furthermore, the values for average variance extracted (AVE) exceed the threshold of 0.5 (Table 4) for convergent validity. Finally, all the composites exhibit discriminant validity, since all the HTMT are below 0.85 (except one of 0.87, below than threshold of 0.9) [78] (Table 4).

**Figure 2:**
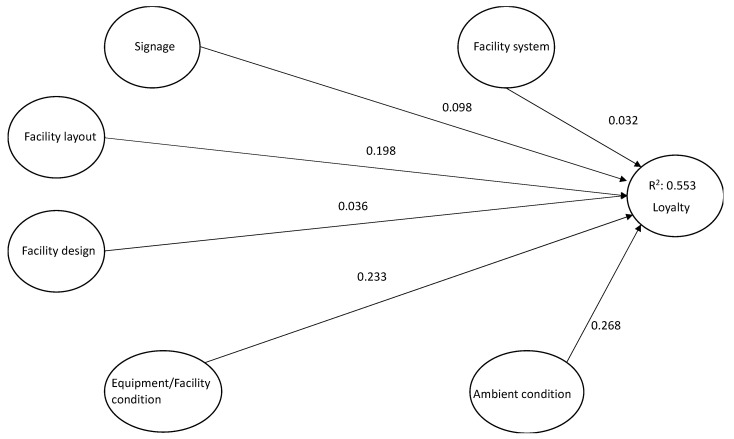
Relationship between constructs.

As to the assessment of the structural model, Hair et al. (2014) comment that the use of bootstrapping (5000 resamples) produces standard errors and confidence intervals to assess the statistical significance of the path coefficients [73]. All the path coefficients in Table 5 and Figure 2 were significant. Our model provided R-squares of 0.55 for loyalty.

**Figure 3:**
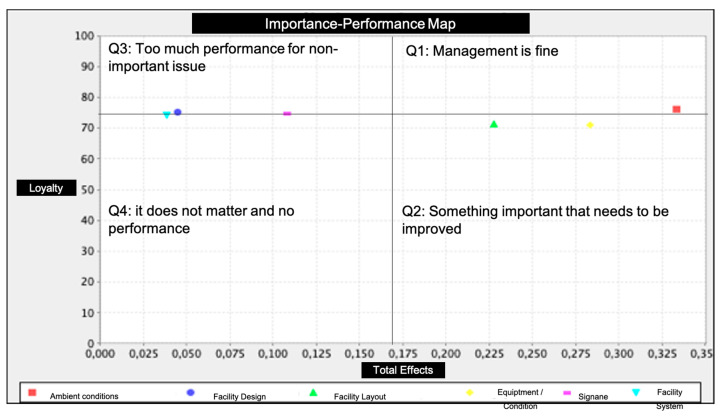
IPMA full data set. Note: Red: ambient condition; Strong blue: facility design; Green: facility layout; Yellow: equipment/facility condition; Pink: signage; Light blue: facility system.

Apart from the PLS-SEM results of a structural model, IPMA helps PLS-SEM results through a four-quadrant diagram as depicted in Figure 3. The vertical axis represents the performance of the attributes from poor performance to good performance. The horizontal axis represents the perceived importance of the attributes from not very important to very important. Following Martilla and James (1977) [14] and Hair et al. (2019) [76], for the importance-map creation, four quadrants are illustrated as Q1 (Management is fine), Q2 (Something important that needs to be improved), Q3 (Too much performance for a non-important issue), and Q4 (it does not matter and no performance). These quadrants are delimited using the mean of performance and mean of importance reported in the table of the IPMA results.

The results are demonstrated in Table 6, while Figure 3 shows the ‘importance-performance map’ of each exogenous latent variable along with its influence on the endogenous latent variable (i.e., loyalty).

**Figure 4:**
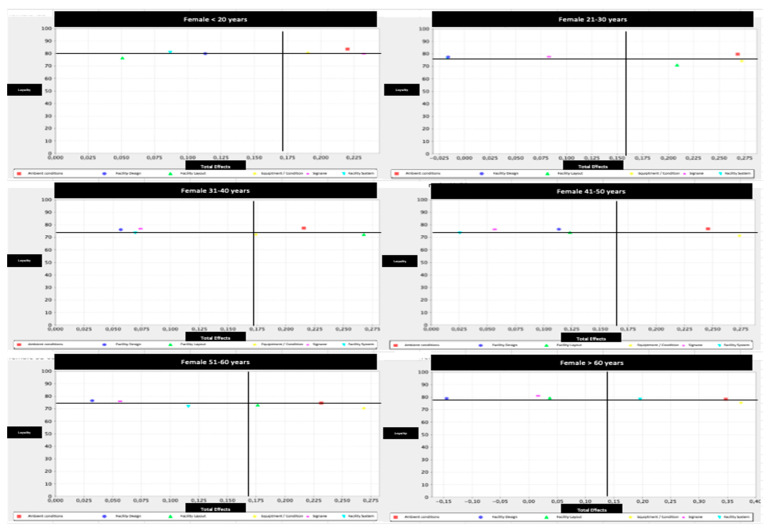
Importance-performance map by female and age group. Note: Red: ambient condition; Strong blue: facility design; Green: facility layout; Yellow: equipment/facility condition; Pink: signage; Light blue: facility system.

**Figure 5:**
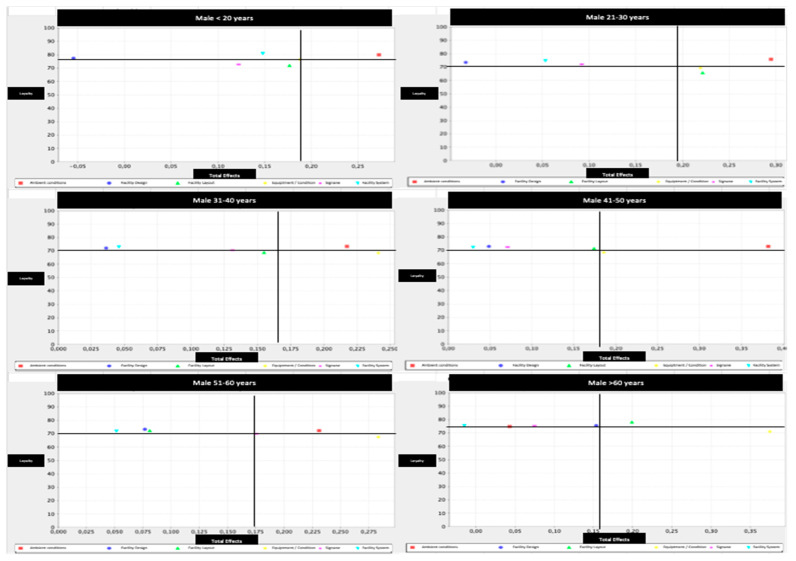
Importance-performance map by male and age group. Note: Red: ambient condition; Strong blue: facility design; Green: facility layout; Yellow: equipment/facility condition; Pink: signage; Light blue: facility system.

Additionally, we decided to distinguish between gender and age, responding to the papers’ call for this special issue. Concretely, we define six age groups (i.e., <20; 21–30; 31–40; 41–50; 51–60; >60) and two sexes (male vs. female), which made a total of 12 categories of fitness center users. Next, we report the IPMA results for these 12 categories and their corresponding map (Table 7, Figure 4 and Figure 5, respectively).

## 5. Discussion

This study analyzed, through the IPMA, the effects of servicescape on customer loyalty in fitness centers. In particular, six aspects were analyzed based on the study by Kim et al. (2016): the ambient conditions, the facility design, the facility layout, the equipment and facility condition, the signages, and the facility system [9]. These factors were in turn related to gender and age to identify what the aspects to be improved were according to the servicescape and sociodemographic variables.

As has been revealed, IPMA is based on standardized regression coefficients (importance) and adds an additional dimension to the analysis that considers the values of the predictor variables, expressed here in terms of a scaled performance index from 0 to 100. The findings have shown the total effect of the independent variables of servicescape on consumer loyalty, calculated together with a scale of latent scores from 0 to 100. These results will add value to those responsible for fitness centers in making decisions to improve customer loyalty [13]. Therefore, our findings confirm previous studies from different sectors that showed that servicescape has a positive relationship with consumer loyalty [29,30,32]. In fact, this research aligns Ong and Yap´s (2017) study in which it was indicated that servicescape had a positive influence on consumers behavioral intentions in fitness centers [8]. However, these authors analyzed the relationship between both variables through four dimensions of servicescape, and the present study has confirmed the positive relationship through six dimensions. In turn, both studies differ by the samples used, having worked in this study with a very high sample of consumers from fitness centers, and analyzing the relationships between servicescape and loyalty through PLS-SEM.

In general terms, having carried out the IPMA the results showed that ambient conditions are the most valuable variable to increase the loyalty of consumers in fitness centers. Precisely, this aspect would be related to music [79], the colors used and the noise level [80], or cleanliness as suggested by Kim et al. (2016) [9] and León-Quismondo et al. (2020) [81]. This fact coincides with the works of Vilnai-Yavetz and Gilboa (2010), Bester (2012), and the study by Lee and Kim (2014) who concluded that a neglected environmental environment could decisively negatively influence a consumer’s loyalty [82,83,84]. However, since the ambient condition already has a high performance index value, there is a low potential for further increase. The best option in terms of the importance and performance ratio seems to be the equipment and facility conditions and the facility layout. In fact, an improvement in the quality of the equipment, the maintenance of the fitness equipment and the own perception of safety of the sports equipment should be favored. Likewise, these results show the need to work on procedures to minimize waiting times in sports services, in turn influencing the design of spaces differentiated by activity levels. Similarly, it was observed that, of all the dimensions analyzed, the facility system versus the facility design were the aspects of minor importance for the sample in general. Specifically, it must be borne in mind that these factors referred to lighting levels, temperature, interior design, decoration, or the correct operation of the heating or air conditioning system.

Regarding the findings on servicescape and the variables of gender and age, the results have shown different aspects to consider to influence the loyalty of sports consumers. This fact is extremely important since authors such as Bernal-García et al. (2018) have already highlighted the need to analyze predictive variables of fidelity in sports consumers according to gender and age [11]. Precisely, men have shown a greater number of aspects where performance indexes can be better and therefore consumer loyalty can be improved. These results corroborate previous studies where women’s perceptions have been more positive in sports services [40,41], although there are also works that indicate the opposite [12]. In fact, in relation to ambient conditions, such as the use of music that is pleasant and adapted to physical activity or the noise level being tolerable, this is a relevant factor for women and men of all the age groups, as concluded by Lee et al. (2019) [30]. Nonetheless, performance indices indicated that there is room for improvement for women aged 51 to 60, and men aged 41 to 60. In this sense, authors such as Bitner (1992) and Chang (2016) reinforce that stimulating environments could favor a positive approach toward the service that in this case would be the fitness center [7,27].

Regarding the facility design, authors such as Whitfield et al. (2014) and Ong and Yap (2017) pointed out the importance of infrastructure and facilities environments to create loyalty in their clients [8,85]. However, this study has shown that it is an aspect of low importance in men and women for consumer loyalty in fitness centers. In turn, the results have shown that the facility layout is possibly the second factor with the greatest margin for improvement in both women and men. This situation is accentuated in men, since the performance indices are below the horizontal axis in the IPMA [76] at ages below 50 years. As for women, the same situation occurs, with the exception of the age groups of under 20, between 41 and 50, and over 60, where this factor is not relevant for fidelity. As stated by Ong and Yap (2017), those responsible for fitness centers should improve functionality and sports spaces for greater consumer comfort [8].

Regarding the equipment and the facility condition, the findings have shown that it is the factor with the greatest margin for improvement in both men and women. In fact, with the exception of women under 20 years of age who are in Q1, all the other age groups are in Q2 [76] and therefore should be improved due to their relationship with consumer loyalty. This fact shows that performance indices, and hence the loyalty of the sports consumer, can be improved. Thus, the results have shown its importance as already indicated by Kim et al. (2016) and Lee et al. (2019) [9,30], in this way influencing the good conditions of the sports facility [8].

This study has also shown the differences between men and women according to the signage factor. Hence, with the exception of women under 20 years of age where it does have a strong influence on fidelity, in the other age groups of women it does not matter. Regarding men, the findings showed margins of improvement in performance indices for men younger than 40 and those between 51 and 60 years old. These results differ from those achieved in fitness centers by Lee et al. (2019) and Ong and Yap (2017) [8,30], who concluded their high need for greater fidelity, as evidenced in other sectors [86].

Regarding the last factor (facility system), the findings showed that only women over 50 are in Q1 [76]. This fact corroborates the results of León-Quismondo et al. (2020) where women, unlike men, do give importance to this [12]. All in all, authors such as Lee et al. (2019) point out that although this factor may not be fundamental to create loyalty in consumers [30], it could at least serve to create a feeling of good service in them and be able to differentiate the centers from the competition.

As to the limitations of the study, this work has been based on the servicescape scale proposed by Kim et al. (2016) that, although it is specific to fitness centers [9], could have deficiencies in relation to decisive factors in consumer perception. Likewise, fidelity was obtained through the behavioral intentions of consumers, which therefore influence subjective behavior. This fact means that the real behavior of consumers could have been modified if objective loyalty variables had been considered [38]. Similarly, having used the IPMA in sports services results in a low comparison with other articles due to the novelty of the analysis, even though it has been used in other sectors. Based on these limitations, the use of other scales and factors that analyze servicescape in sports facilities and which, in turn, is analyzed in different sports spaces, are proposed as future lines of research. Likewise, it is necessary to use objective behavioral variables in future investigations to identify aspects that could influence consumer loyalty. Finally, other researchers are urged to use IPMA for a better understanding of aspects that could be evaluated and improved by managers, in favor of sports consumers’ greater loyalty.

## 6. Practical Implications

The study is almost certainly the largest sample carried out on servicescape in fitness centers. Its results are very interesting and can be extrapolated when it comes to proposing strategies about servicescape in fitness centers. In fact, although the results have shown differences in terms of gender and age, it is proposed to focus efforts on facility layout, equipment, and facility conditions. Specifically, it is recommended that managers improve the perception of the equipment’s functionality, and the perception of waiting times (such as the enrollment process, attention to prescribe a training program, or any specific customer service process). Another aspect to improve is the feeling of spaciousness in sports spaces. In fact, due to the post-Covid-19 situation, this aspect could recently have been improved due to the increase in the distance between the sports equipment, which would provide a greater sense of spaciousness. Finally, the managers of the fitness centers should also focus their efforts on the continuous maintenance of the equipment as well as safety strategies for sports consumers.

## 7. Conclusions

The objective of this study was to analyze the relationship between servicescape and the loyalty of fitness center consumers, this being examined by gender and age with the IPMA. In this sense, the results of this research show interesting results in different lines. Our findings demonstrate the relationship between servicescape and loyalty in fitness centers consumers. The results have shown the high importance that consumers give to tangibility in fitness centers, from which the managers of the sports facilities must manage and adapt correctly.

In general, the aspects with room for improvement and affecting consumer loyalty in fitness centers were the equipment and the facility conditions, and the facility layout. In this sense, fitness centers must improve the safety, maintenance and quality of sports equipment. They must also get a better design of sports spaces with which consumers are more comfortable and perceive less waiting time to perform physical activities.

In relation to gender and age, the aspects which could be improved were to a greater extent the equipment and facility condition in women over 21 years old, and the facility layout for women between 21 to 40 years old and 51 to 60 years old. They also have room to improve the signage for women under 21 years of age. That is, the signage must be helpful. Regarding the perception of women over 51 years of age, ambient conditions such as cleanliness, noise or the environment could also have room for improvement. Regarding men, the aspects with the highest performance margins were the equipment and facility conditions in all the age groups, the facility layout in men from 21 to 30 years, and the signage in men from 51 to 60 years. In conclusion, there is still room for increasing consumer loyalty through aspects related to servicescape.

## Figures and Tables

**Table 1:** Importance-Performance Matrix Analysis (IPMA) studies.

Authors	Sector/Context	Principal Findings Related	Sample
Tailab [55]	Bank	The study indicates that managers who use the IPMA to prioritize their financial decisions will obtain useful conceptual insights and are unlikely to be misled.	140 banks
Ting, Yahya & Tan [56]	Education	The IPMA shows that the importance and performance of domain knowledge are high in contributing to sustainability entrepreneurship.	121 students
Ebrahimi, Hajmohammadi & Tan [57]	Tourism	The IPMA indicates that place image had the highest importance, but the lowest performance.	135 users
Wook, Ismail & Yusop [58]	Education	The findings revealed through the IPMA that perceived usefulness is the most important antecedent, followed by perceived ease of use, and optimism.	211 students
Groß [59]	Mobile	Thanks to the IPMA, this study provides new insights into the acceptance and profile of m-shoppers.	734 customers
Palos-Sánchez, Martin-Velicia & Saura [60]	Internet users	The IPMA showed the most important attributes for Internet search engine developers.	445 users
Carranza, Díaz & Martín-Consuegra [61]	Restaurants	The IPMA indicate that service quality is one of the three most-valued attributes among those examined in fast-food restaurants studies.	456 customers
Reyes-Menéndez, Palos-Sánchez, Saura & Martín-Velicia [62]	Restaurants companies	The IPMA indicated that Wi-Fi has the highest valuation, although it is the one that obtains the least performance. It is in this construct that improvements in performance must be made.	117 customers
Ramayah, Chiun, Rouibah & May [63]	Online banking	The IPMA identified that the two most important variables in the use of Internet banking are perceived ease of use and perceived ease of use.	239 customers
Rigdon, Ringle, Sarstedt & Gudergan [64]	Utilities and hotels	The IPMA illustrates differences between three segments of customers and provides the basis for managerial implications.	5398 customers

**Table 2:** Sample characteristics.

Question	Answer	Women		Men		Total	
		*n*	%	*n*	%	*n*	%
Weekly frequency	Less than 1 time/week	149	2.54%	100	2.22%	249	2.40%
	Once/week	242	4.13%	143	3.17%	385	3.71%
	Twice/week	1369	23.35%	646	14.34%	2015	19.43%
	Three times/week	2423	41.32%	1813	40.25%	4236	40.86%
	Four more times/week	1681	28.67%	1802	40.01%	3483	33.59%
	Total	5864	100.00%	4504	100.00%	10,368	100.00%
Previous fitness experience	No previous experience	1558	26.57%	899	19.96%	2457	23.70%
	Yes, in the current fitness center	259	4.42%	187	4.15%	446	4.30%
	Yes, in another fitness center	4047	69.01%	3418	75.89%	7465	72.00%
	Total	5864	100.00%	4504	100.00%	10,368	100.00%
Training prescription	Fitness staff	758	12.93%	434	9.64%	1192	11.50%
	Friend	296	5.05%	203	4.51%	499	4.81%
	Personal trainer	247	4.21%	220	4.88%	467	4.50%
	Fitness App	202	3.44%	130	2.89%	332	3.20%
	Myself	3840	65.48%	3303	73.33%	7143	68.89%
	Others	521	8.88%	214	4.75%	735	7.09%
	Total	5864	100.00%	4504	100.00%	10,368	100.00%

**Table 3:** Indicator loadings.

Construct	Indicator	Loadings
Loyalty	I would sign up for this fitness center if I unsubscribed (FI1)	0.895
	I will make positive comments to a friend about the programs and services of this fitness center (FI2)	0.954
	If you ask me, I will recommend this fitness center (FI3)	0.967
Signage	The signs used are helpful (SC1)	1
Equipment/Facility condition	The equipment used is always in good working condition (SC10)	0.894
	Fitness center is well equipped with surrounding facilities (lounge, concession) (SC11)	0.587
	The facilities and equipment are safe (SC12)	0.877
	Physical facilities are well maintained (SC13)	0.903
	The equipment used is of high quality (SC9)	0.879
Ambient Condition	The background noise level at fitness center is acceptable (SC14)	0.854
	Fitness center is kept clean (SC15)	0.800
	Fitness center’s atmosphere is comfortable (SC16)	0.885
	The music used in fitness center makes workout environment a more enjoyable place (SC17)	0.812
Facility System	Lighting levels are comfortable (SC18)	0.789
	Temperature and humidity are comfortable (SC19)	0.913
	Air quality is acceptable (SC20)	0.917
	Heating, Ventilation and Air-conditioning (HVAC) system are well maintained (SC21)	0.898
Facility Layout	Fitness center is expansive and large in scale (SC2)	0.869
	Fitness center is designed for all levels of ability (SC3)	0.838
	Fitness center have more than enough space for me to be comfortable (SC4)	0.909
	Fitness center is designed to minimize my waiting time (SC5)	0.860
Facility Design	The buildings’ exterior layout is pleasing (SC6)	0.862
	The buildings’ interior layout is pleasing (SC7)	0.944
	The buildings in fitness center are decorated in an attractive fashion (SC8)	0.934

FI: Future Intentions; SC: Servicescape.

**Table 4:** Reliability and validity of construct measurement.

Composites	Cronbach’s Alpha (α)	Dijkstra- Henseler’s Rho (ρ_A_)	Composite Reliability (ρ_c_)	Average Variance Extracted	Maximum HTMT ^a^
Ambient Condition	0.859	0.870	0.904	0.703	0.838
Facility Design	0.901	0.914	0.938	0.836	0.741
Facility Layout	0.892	0.894	0.925	0.756	0.869
Equipment/Facility Condition	0.887	0.912	0.919	0.700	0.778
Signage	1	1	1	1	1
Facility System	0.902	0.902	0.932	0.776	0.881
Loyalty	0.933	0.938	0.957	0.882	0.839

^a^ HeteroTrait-MonoTrait ratio of correlations for discriminant validity.

**Table 5:** Construct effects on endogenous variables (incl. lower and upper limits of 95% confidence interval).

Relationships	Original Sample (O)	Average of the Sample (M)	5.0%	95.0%
Ambient Condition -> Loyalty	0.258	0.258	0.233	0.282
Facility Design -> Loyalty	0.036	0.036	0.010	0.061
Facility Layout -> Loyalty	0.198	0.198	0.176	0.220
Equipment/Facility Condition -> Loyalty	0.233	0.233	0.208	0.257
Signage -> Loyalty	0.096	0.096	0.076	0.115
Facility System -> Loyalty	0.032	0.032	0.012	0.053

**Table 6:** IPMA results full data set.

Latent Variable	Loyalty	
	Total Effect (Importance)	Index Value (Performance)
Ambient Condition	0.333	76.082
Facility Design	0.045	75.254
Facility Layout	0.228	71.483
Equipment/Facility Condition	0.283	71.073
Signage	0.108	74.762
Facility System	0.039	74.108
Mean	0.173	73.794

**Table 7:** IPMA results by sex and age group.

**Male**	**Male < 20**		**Male 21–30**		**Male 31–40**		**Male 41–50**		**Male 51–60**		**Male > 60**	
	I	P	I	P	I	P	I	P	I	P	I	P
Ambient Condition	0.379	79.875	0.406	75.722	0.295	73.321	0.465	72.869	0.278	72.187	0.053	74.724
Facility Design	−0.066	77.337	−0.042	73.486	0.047	72.150	0.061	73.042	0.098	73.391	0.191	75.637
Facility Layout	0.201	71.866	0.251	65.816	0.183	68.654	0.200	71.454	0.098	72.403	0.248	78.008
Equipment/Facility Condition	0.253	76.334	0.271	69.552	0.299	68.443	0.220	68.395	0.333	67.405	0.435	71.066
Signage	0.121	72.717	0.104	72.171	0.149	70.668	0.079	72.335	0.189	70.057	0.084	75.342
Facility System	0.199	80.836	0.064	74.808	0.057	73.011	0.034	72.337	0.061	71.994	−0.018	75.556
**Female**	**Female < 20**		**Female 21–30**		**Female 31–40**		**Female 41–50**		**Female 51–60**		**Female > 60**	
	I	P	I	P	I	P	I	P	I	P	I	P
Ambient Condition	0.309	83.519	0.361	79.562	0.286	77.434	0.304	76.699	0.277	74.542	0.365	78.295
Facility Design	0.124	79.865	−0.020	77.393	0.072	76.187	0.139	76.520	0.040	76.632	−0.149	79.046
Facility Layout	0.054	76.542	0.231	71.142	0.307	72.220	0.142	74.070	0.203	72.865	0.038	79.310
Equipment/Facility Condition	0.227	80.538	0.336	74.149	0.219	72.156	0.323	71.195	0.317	70.522	0.403	75.622
Signage	0.208	79.821	0.095	77.759	0.087	76.945	0.064	76.340	0.066	75.964	0.017	80.978
Facility System	0.098	80.969	−0.021	76.536	0.082	73.634	0.030	73.468	0.132	71.903	0.200	78.412

I: Importance; P: Performance.
